# Phylogenetic analysis of West Nile Virus in Maricopa County, Arizona: Evidence for dynamic behavior of strains in two major lineages in the American Southwest

**DOI:** 10.1371/journal.pone.0205801

**Published:** 2018-11-26

**Authors:** Crystal M. Hepp, Jill Hager Cocking, Michael Valentine, Steven J. Young, Dan Damian, Kimberly E. Samuels-Crow, Krystal Sheridan, Viacheslav Y. Fofanov, Tara N. Furstenau, Joseph D. Busch, Daryn E. Erickson, Ryan C. Lancione, Kirk Smith, James Will, John Townsend, Paul S. Keim, David M. Engelthaler

**Affiliations:** 1 School of Informatics, Computing, and Cyber Systems, Northern Arizona University, Flagstaff, Arizona, United Sates of America; 2 The Pathogen and Microbiome Institute, Northern Arizona University, Flagstaff, Arizona, United States of America; 3 Translational Genomics Research Institute, Flagstaff, Arizona, United States of America; 4 Maricopa County Environmental Services Department Vector Control Division, Phoenix, Arizona, United States of America; 5 Maricopa County Environmental Services Department Office of Enterprise Technology, Phoenix, Arizona, United States of America; University of Reunion Island, RÉUNION

## Abstract

West Nile Virus (WNV) has been detected annually in Maricopa County, Arizona, since 2003. With this in mind, we sought to determine if contemporary strains are endemic to the county or are annually imported. As part of this effort, we developed a new protocol for tiled amplicon sequencing of WNV to efficiently attain greater than 99% coverage of 14 WNV genomes collected directly from positive mosquito pools distributed throughout Maricopa County between 2014 and 2017. Bayesian phylogenetic analyses revealed that contemporary genomes fall within two major lineages; NA/WN02 and SW/WN03. We found that all of the Arizona strains possessed an amino acid substitution known to be under positive selection, which has arisen independently at least four times in Arizona. The SW/WN03 strains exhibited transient behavior, with at least 10 separate introductions into Arizona when considering both historical and contemporary strains. However, NA/WN02 strains are geographically differentiated and appear to be endemic in Arizona, with two clades that have been circulating for four and seven years. This establishment in Maricopa County provides the first evidence of local overwintering by a WNV strain over the course of several years in Arizona. Within a national context, the placement of eleven contemporary Arizona strains in the NA/WN02 lineage indicates while WNV first entered the northeastern United States in 1999, the most ancestral extant strains of WNV are now circulating in the American southwest.

## Introduction

**Fig 1:**
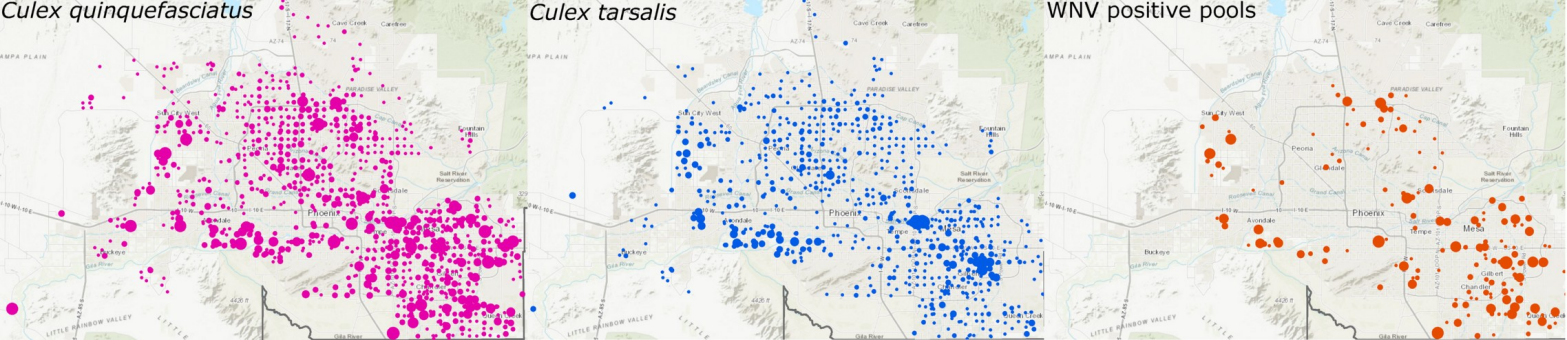
Distribution of WNV and its vectors across Maricopa County in 2017. The dots represent the count of individual female *Culex quinquefasciatus* (left), *Culex tarsalis* (center), or WNV positive mosquito pools (right) at each of 787 carbon dioxide traps distributed primarily throughout the urban portion of the county. Larger circles indicate a higher density at a particular trap. The USGS Topo was used as a basemap for this figure (USGS The National Map: National Boundaries Dataset, National Elevation Dataset, Geographic Names Information System, National Hydrography Dataset, National Land Cover Database, National Structures Dataset, and National Transportation Dataset; U.S. Census Bureau—TIGER/Line; HERE Road Data. Data Refreshed July, 2017). The final map was created using ArcGIS software by Esri. ArcGIS and ArcMap are the intellectual property of Esri and are used herein under license. Copyright Esri. All rights reserved.

The first human cases of West Nile Virus (WNV) were identified in New York City during the summer of 1999. This mosquito-borne virus belongs to the Japanese encephalitis complex (genus *Flavivirus*) and became well established throughout the United States by 2004. Almost two decades later, WNV is still the most important arbovirus nationwide, causing 96% of domestic arboviral diseases reported to the Centers for Disease Control and Prevention (CDC) in 2016 [1, 2]. The remaining cases are caused by: La Crosse virus, St. Louis encephalitis virus, Jamestown Canyon virus, Powassan virus, eastern equine encephalitis virus, unspecific California serogroup virus, and Cache Valley virus [1, 2]. From 2007–2016 there have been 22,111 human cases, 11,668 which were neuroinvasive, and 1,054 deaths associated with WNV infections reported to the CDC [3]. Of those cases, nearly 24% (n = 5,282) have occurred in the southwestern states of Nevada, California, Utah, and Arizona [1]. Maricopa County, the most populous county in Arizona, and the 4^th^ most populous county in the United States, detected its first WNV-positive bird (*Passer domesticus–*House sparrow) in September 2003. A positive mosquito pool was detected one week later, followed shortly thereafter by the first autochthonous human case in November of that same year. Human WNV cases within Maricopa County peaked dramatically in 2004 with 355 human cases and 14 deaths [4]. A lesser spike in human infections occurred in 2010, with 155 reported cases. While WNV is not presently affecting the human population of Maricopa County to the extent it did in 2004 and 2010, the virus has reliably infected humans in the area each year since its first detection. In 2017, there were 110 confirmed or probable human cases in Arizona, with 97 (88%) of those being neuroinvasive cases. The Vector Control District of Maricopa County Environmental Services detected WNV in 221 positive mosquito pools (71% were *Culex quinquefasciatus* pools) of 12,977 tested across the Phoenix Metropolitan area [5] (Fig 1). Given the annual prevalence of WNV in Maricopa County, the purpose of this study was to answer the overarching question: Are Maricopa County WNV populations endemic residents or are they reintroduced from other foci annually?

## Materials and methods

### Sample collection

**Fig 2:**
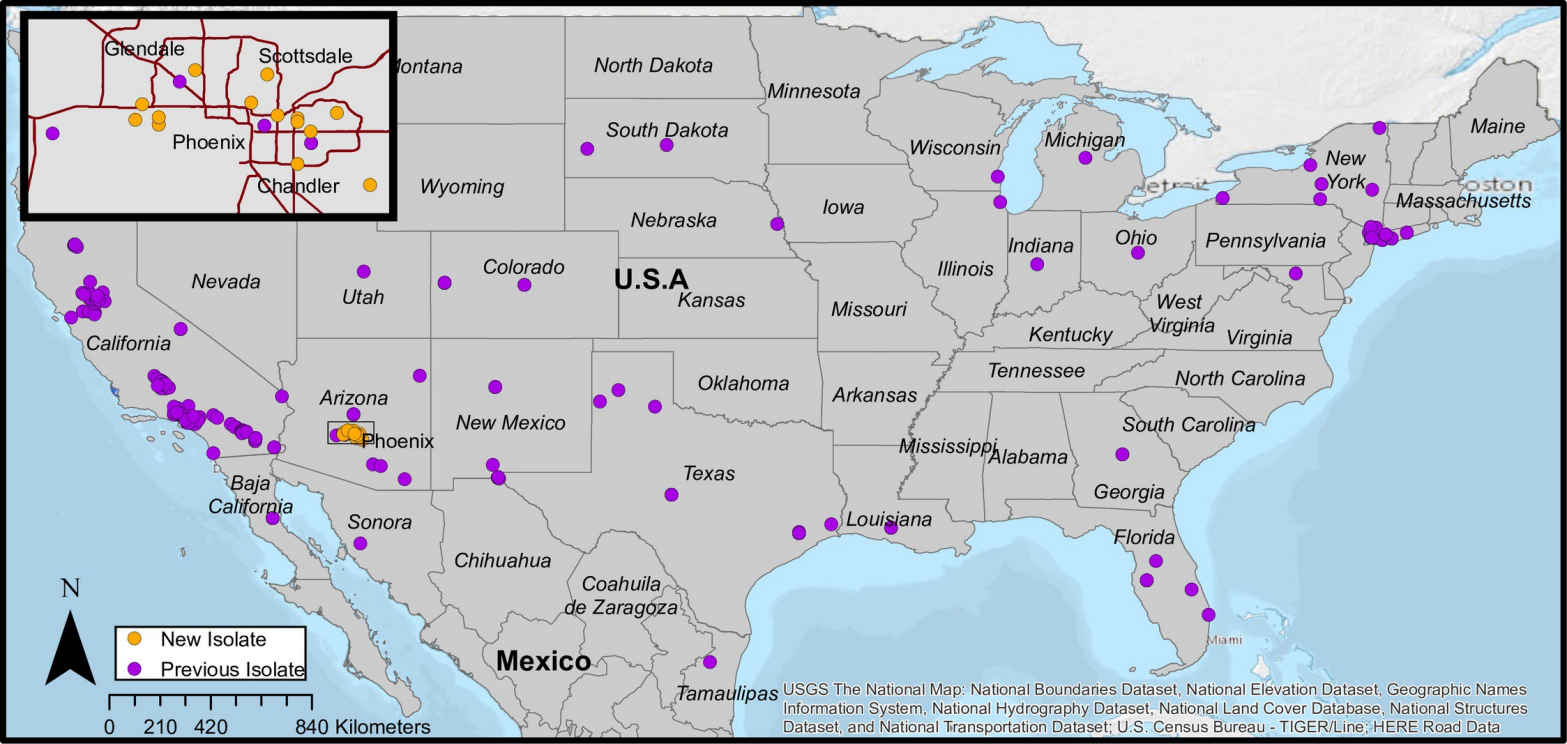
Geographic distribution of WNV strains included in the phylogenetic analysis, except for the New York strains published in Ehrbar et al., [17]where latitude and longitude coordinates were not available. Purple points indicate publicly available strains while orange points (inset map area shown with rectangle) indicate strains that were sequenced as part of this study. Additional metadata can be found in S1 Table. The USGS Topo was used as a basemap for this figure (USGS The National Map: National Boundaries Dataset, National Elevation Dataset, Geographic Names Information System, National Hydrography Dataset, National Land Cover Database, National Structures Dataset, and National Transportation Dataset; U.S. Census Bureau—TIGER/Line; HERE Road Data. Data Refreshed July, 2017). The final map was created using ArcGIS software by Esri. ArcGIS and ArcMap are the intellectual property of Esri and are used herein under license. Copyright Esri. All rights reserved.

The Maricopa County Environmental Services Department Vector Control Division conducts year-round mosquito surveillance and abatement activities throughout Maricopa County. As part of their general protocol, mosquitoes are collected once weekly from 787 routine carbon dioxide trap locations distributed throughout the Phoenix metropolitan area (Fig 1). In the case where traps are set up on private property, Maricopa County Environmental Services Department Vector Control Division receives permission from the landowner to set traps. Permissions are not required for the agency to set traps in locations on public lands, as they are part of the county government. The field studies did not involve endangered or protected species. Collections are subsequently sorted by species and sex. Up to 5 pools, each composed of up to 50 female *Cx*. *quinquefasciatus* or *tarsalis* mosquitoes, are individually tested for WNV, using the protocol described in Lanciotti et al. [6]. We selected 14 WNV positive mosquito pools, distributed geographically (Fig 2) and temporally (from 2014 to 2017, S1 Table), for whole genome tiled amplicon sequencing using a novel protocol, based on the method of Quick et al. [7] developed for Zika virus sequencing.

### Sample processing, a new WNV tiled amplicon protocol, and next generation sequencing

Prior to transporting the samples, an equal part of DNA/RNA Shield^TM^ 2X Concentrate (Zymo Research) was added to each mosquito pool to stabilize the RNA and inactivate WNV. The pools consisted of mosquitos in approximately 1.3 ml of TE buffer (Invitrogen, AM9858). All mosquito pools were stored at -80 degrees Celsius prior to extractions. Both DNA and RNA were extracted from the pools in a Biosafety Cabinet using the Quick DNA/RNA Pathogen Miniprep^TM^ kit (Zymo Research). RNA was reverse transcribed into cDNA using the protocol as described in Quick et al. [7], and the product was stored at -20 degrees Celsius.

The Primal Scheme primer designer software [7] was used to design the multiplex PCR primers for our tiled amplicon sequencing protocol. A total of 41 primer pairs with attached universal tails, split into two pools, were used to amplify regions averaging 372 bases in length, covering the NY99 (NC_009942.1) genome positions 8–10877 (S2 Table). The preparation of the tiled amplicons was carried out as described by Colman et al. [8]. For the initial amplification of specific regions of the WNV genome, each sample was prepared with two pools of primers. Each of the two PCRs per sample consisted of 12.5 μl of KAPA 2G Fast Multiplex PCR Mastermix (Kapa Biosystems, Wilmington, MA), primers from pool 1 or 2 for a final concentration of 0.2 μM each primer, and 2.5 μl of cDNA. The PCR was performed as follows: 3 minutes of denaturation at 95°C, 30 cycles of 95°C for 15 seconds, 60°C for 30 seconds, 72°C for 1 minutes, and a final extension of 72°C for 1 minute. The PCR products were cleaned using 1X Agencourt AMPure XP beads (Beckman Coulter, Indianapolis, IN). Illumina’s sample-specific index and sequencing adapters were added during a second PCR utilizing the universal tail-specific primers. This reaction was prepared with 12.5 μl of 2X Kapa HiFi HotStart Ready Mix (Kapa Biosystems), 400 nM of each forward and reverse indexed primer, and 2 or 4 μl the cleaned and amplified WNV product. This PCR was performed as follows: 98°C for 2 minutes, 6 cycles of 98°C for 30 seconds, 60°C for 20 seconds, 72°C for 30 seconds, and finally 72°C for 5 minutes. The indexed PCR products were again cleaned with 1X Agencourt AMPure XP beads (Beckman Coulter). The amplicon libraries from each WNV sample were quantified using the Kapa Library Quantification kit (Kapa Biosystems) and pooled in equal concentrations. Sequencing was carried out on the Illumina Miseq sequencing platform, using a v2 500 cycle kit.

### Sequence data processing

Sequencing reads were aligned to the NY99 Lineage 1 WNV genome (NC_009942.1) using samtools [9] and Bowtie2 [10]. Alignments were visualized in the Integrative Genomics Viewer (IGV), and all sequenced positions had at least 100x coverage [11, 12]. Consensus sequences were exported from IGV, where at least 80% of reads at a position had to have the majority allele for a position to be called an A, T, G, or C. For sites where the majority allele was not represented in at least 80% of the reads at that position, sites were coded according to the International Union of Pure and Applied Chemistry (IUPAC) nucleotide codes. The 14 new consensus genomes and associated metadata were deposited into Genbank using Bankit (Accession numbers: MG004528-MG004541).

### Phylogenetic analysis with an incorporated timescale

Using MUSCLE in MEGA7.0 [13], we aligned a total of 246 genomes, including: i) newly sequenced genomes from this study (n = 14); ii) United States-based whole genomes that were included in Pybus et al. [14] (n = 104); iii) Arizona-based genomes that were published in Plante et al. [15] (n = 3); iv) Southern California-based genomes published in Duggal et al. [16] (n = 112); and v) New York-based genomes [17] (n = 13) (S1 Table). Genomes were selected based on availability of sample metadata, including time and geolocation of collections, except for the New York genomes from Ehrbar et al., [17], where exact geolocation coordinates were not available. To determine if the WNV genomes in those data exhibited a strong molecular clock signal, we constructed a neighbor joining tree in MEGA7.0 [13] and uploaded the newick file, with associated collection dates into TempEst [18]. The coefficient of determination revealed that nearly 90% (R^2^ = 0.8871) of the variation in root to tip distance can be explained by time, indicating that a molecular clock analysis would be appropriate for the dataset.

We employed a Bayesian molecular clock method implemented in the BEAST v1.8.4 [19] software package to estimate evolutionary rates for WNV, as well as divergence times for Arizona lineages. Substitution model selection was carried out in MEGA 7.0.9 [13] for the 246 genomes included in our dataset. The corrected Akaikes’s Information Criterion and Bayesian Criterion results indicated that the General Time Reversible model with incorporation of a gamma distribution of among-site rate variation best fit the dataset. To determine the best fitting clock and demographic model combinations for these data, the generalized stepping stone marginal likelihood estimator [20] was employed to compare the Strict or Uncorrelated Lognormal (UCLN) [21] clock models combined with the Constant, Exponential, Gaussian Markov Random Field Bayesian Skyride [22], or the Bayesian Skygrid demographic [23] models. The eight model combinations were each iterated in duplicate, for 100,000,000 generations, where Markov chains were sampled every 10,000 generations. We found that the UCLN clock and Bayesian Skygrid combination outperformed the other seven combinations (S3 Table). Using the UCLN Bayesian Skygrid model, we ran two additional chains for 100,000,000 generations, sampling every 10,000, and found convergence within and among chains using Tracer v1.6 [24]. We used LogCombiner to merge the four different chains, discarding the first 10% as burn in (10,000,000 generations per chain), and then resampled every 40,000 generations. The resulting file was input to TreeAnnotator to produce a maximum clade credibility tree, and then visualized using FigTree v1.4.3 [25].

### Defining WNV lineages

**Fig 3:**
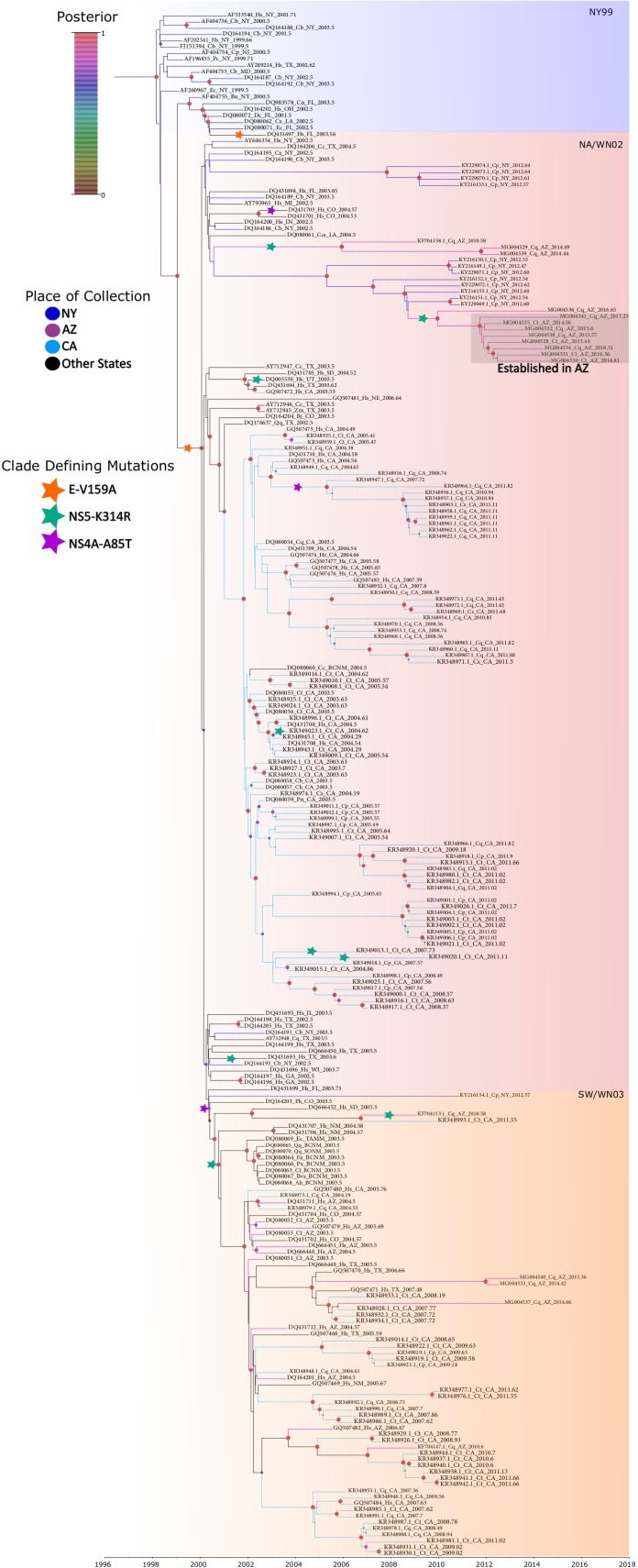
Tip-labelled maximum clade credibility phylogenetic tree reconstructed from 246 nationally distributed WNV genomes. Each tip consists of the accession number, vector or host where the strain was derived, two letter state code (or 4 letter code for location in Mexico), and the date of sampling to the nearest hundredth of a year. The gradient squares indicates the phylogenetic position of the NY99 (blue), NA/WN02 (red), and SW/WN03 (orange) genotypes. NA/WN02 and SW/WN03 strains are defined by the E-V195A substitution and the SW/WN03 genotype is defined by the NS4A-A85T substitution. Clade-defining substitutions, as well as convergent substitutions, are denoted by stars. Posterior probabilities are represented by the size (larger circles have higher values) and color (defined by the color legend) of circles at each node.

The three major lineages of WNV in the United States have classically been defined by two amino acid substitutions (E-V159A [26, 27] and NS4A-A85T [28]) within the polyprotein, and we have indicated the locations of these substitutions on the reconstructed phylogeny (Fig 3 and S1 Table). Members of the NY99 lineage, the most ancestral lineage of WNV that entered North America, possess an valine at position 159 of the envelope protein [26, 27], while nearly all isolates collected after 2002 have an alanine at that position, and are part of both the North American/WN02 (NA/WN02) and distal Southwestern/WN03 (SW/WN03) lineages. The entire SW/WN03 genotype (Groups 1–5 defined by McMullen et al. [28]) is defined by the NS4A-A85T substitution. The more distal SW/WN03 taxa (Groups 3–5 defined by McMullen et al. [28]) additionally possess the NS5-K314R substitution, which also independently arose in a clade of Southern California strains residing within the NA/WN02 lineage (S1 Table and Fig 3) [16]. Phylogenetically ordered amino acid alignments of the envelope (E) and two nonstructural proteins (NS4 and NS5) as well as a nucleotide alignment of the coding region have been included as supplementary files (S1, S2, S3 and S4 Files, respectively).

## Results and discussion

In an effort to better understand the recent dynamics of West Nile Virus circulation in the Phoenix metropolitan area of Maricopa County, Arizona, we sequenced 14 genomes and evaluated them within a nationwide context to estimate: 1) when WNV was first introduced into Maricopa County, AZ, 2) how many distinct WNV introductions have occurred, 3) if contemporary strains belong to lineages that have become endemic in Maricopa County, and 4) the temporal span of such establishment.

The phylogeny revealed that WNV strains in Arizona have been genetically diverse over time, and are represented in both major lineages (NA/WN02 and SW/WN03) still circulating in the United States (Fig 3). The placement of Maricopa County strains within the NA/WN02 and SW/WN03 lineages was first described in Plante et al. [15], and in agreement, we find that 11 of the genomes sequenced here belong to the NA/WN02 lineage, while 3 others cluster within the SW/WN03 lineage.

Maricopa County strains interspersed within the SW/WN03 lineage exhibit polyphyletic clustering, and are most closely related to strains collected in California, Colorado, Texas, and New Mexico. Several of the most basal clusters diverging in 2002 are characterized by short branches, implying that the lineage rapidly expanded at that time. The lack of geographic structure at the state level, aside from several strains collected in southern California [16], indicates that the lineage is circulating across long distances in the southwestern United States. The SW/WN03 lineage was the first to be introduced into Arizona, during 2002 (mean estimate: 2002.81, 95% CI: 2002.66–2003.17) (Fig 3, clade including DQ080052, GQ507479, DQ080053, DQ666451, DQ666448), and this initial introduction represents the longest period of time that any of the SW/WN03 introductions persisted in Arizona. After this initial introduction to Arizona, the SW/WN03 lineage was introduced, or perhaps reintroduced, into Texas, California, and New Mexico, and strains from Texas and California appear to have reseeded Arizona. However, each of these reintroductions are represented by only one or two strains, indicating that the SW/WN03 lineage does not appear to have a stronghold in Arizona. The contemporary strains sequenced in this study (MG004533, MG004540, and MG004537) represent two introductions that are part of a small clade including strains from Southern California and Texas. MG004540 was collected in 2015, suggesting that this lineage may still be in circulation in the county. We are in the process of sequencing additional strains from 2014–2018 and will further investigate the presence of the SW/WN03 lineage in Arizona.

The NA/WN02 lineage was first detected in Arizona in 2010 [15], and the strain representing the first detection (KF704158.1) clusters monophyletically with two strains collected in 2014 (MG004529 and MG004539) (Fig 3). The estimate of time to most recent common ancestor (TMRCA) of this clade indicates that the initial introduction of NA/WN02 occurred in 2007 (mean estimate: 2007.31, 95%CI: 2005.86–2009.23), three years prior to the detection of KF704158.1. While we find that strains from Arizona are dispersed throughout the tree, nine of the fourteen strains sequenced as part of this study clustered monophyletically, with a most recent common ancestor entering Arizona in 2011 (mean estimate: 2011.42, 95%CI: 2010.63–2012.7). However, there is a long branch separating MG004536 from the other eight strains, and data presented on Nextstrain [29] indicate that MG004536 is actually part of a separate entrance from Texas. The remaining monophyletic clade (mean estimate of TMRCA: 2013.54, 95%CI: 2012.98–2014.01), which includes eight strains collected from 2014 through 2017 (Fig 2, grey box), is nested within a paraphyletic clade of New York strains that were collected in 2012. This monophyly and node dating indicates a recent Arizona introduction of a WNV population recently detected in New York, which was likely more gradational than our current dataset can demonstrate due to the complexities of avian dispersal and incomplete national genomic surveillance. However, within the available national context, the placement of eleven contemporary Arizona strains in the NA/WN02 lineage indicates while WNV first entered the northeastern United States in 1999, the most ancestral extant strains are now circulating in the southwest.

**Fig 4:**
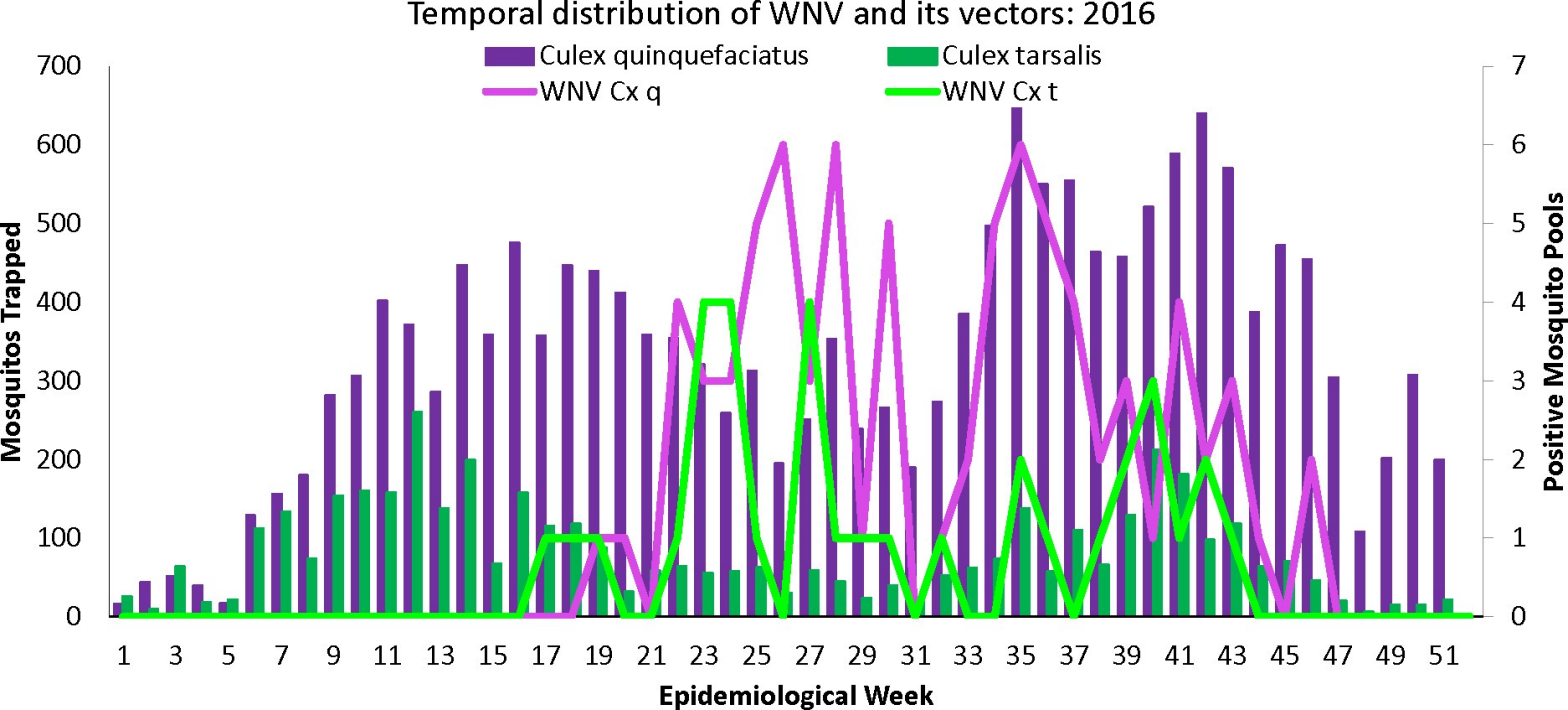
Distribution of *Culex* mosquitoes and WNV positive pools in Maricopa County by epidemiological week in 2016. The first y-axis represents the number of *Culex quinquefasciatus* (purple bars) and *Culex tarsalis* (green bars) individuals trapped each epidemiological week. The second y-axis represents the number of WNV positive *Culex quinquefasciatus* (purple line) and WNV positive *Culex tarsalis* (green line) mosquito pools trapped each epidemiological week.

The monophyletic nature of the eight tightly clustered contemporary Maricopa County NA/WN02 strains indicates that, at least for this lineage, there is most likely a mechanism that allows for viral overwintering in resident birds and/or *Culex* mosquitoes living in Maricopa County. Komar et al. [30] performed an extensive study in 2010 to identify Arizona-resident avian hosts of WNV. They found that communal roosting house sparrows (*Passer domesticus*), house finches (*Haemorhous mexicanus*), great-tailed grackles (*Quiscalus mexicanus*), and mourning doves (*Zenaida macroura*) account for the greatest proportion of resident bird infections. In addition to highly competent resident bird species in Maricopa County, both *Cx*. *tarsalis* and the more abundant *Cx*. *quinquefasciatus* vectors are present year-round in Maricopa County (Fig 4). However, more extensive surveillance is needed during the winter season to better understand which mechanisms support overwintering. Although WNV has not historically displayed strong geographic clustering [31], monophyletic clustering of the endemic Arizona strains is similar to clustering of strains collected from Southern California [16]. Collectively, these studies indicate that the American southwest presents a suitable habitat for WNV to establish and persist across multiple years.

Perhaps the most intriguing result of this study is that despite the diversity of Arizona strains over time, all carry the amino acid substitution of NS5-K314R. This substitution appears to have arisen in 2001 (mean estimate: 2001.41, 95%CI: 2001.05–2002.18, Fig 3), in the SW/WN03 lineage, and then independently arose on three separate occasions in Arizona strains; twice in the NA/WN02 lineage and one additional time in the SW/WN03 lineage (Fig 3). This substitution has also independently occurred in other southwestern strains from Texas, California, and Utah (Fig 3), but several representatives from these states do not contain the substitution. Given that previous selection analyses have identified this substitution as being positively selected [28], we hypothesize that this substitution is important for adaptation to the Arizona ecosystem, and is at least beneficial to survival in other parts of the southwest United States.

It is important to note three caveats to this study. First, we used the consensus sequence approach outlined in the methods; with this approach, it is possible that we are not capturing other strains circulating at lower levels within mosquito pools. Second, the short branch lengths within the SW/WN03 lineage coupled with low posterior probabilities and limited sampling may indicate that rather than a short lived first introduction, the lineage is cryptically circulating in Arizona at low levels. Third, and related to the second point, our results are constrained by the availability of WNV isolates and strains at the time of the study. As with any phylogenetic analysis of infectious agents, we expect that additional sequencing of strains from the southwestern U.S. will add context and clarification to some of the results presented here.

The overarching goal of this study was to determine if strains of WNV currently circulating in Maricopa County are endemic or annually imported. The 14 contemporary strains sequenced here reveal that both NA/WN02 and SW/WN03 lineages have been recently detected in Maricopa County. Interestingly, only 3 of the contemporary strains fall within the SW/WN03 lineage, despite the more frequent membership of Arizona strains in this lineage historically. In contrast, while few AZ representatives have clustered within the NA/WN02 lineage in the past, 11 of the contemporary strains sequenced as part of this study clustered within that lineage. Nine of the strains from the NA/WN02 lineage clustered monophyletically, and we estimate that strains from this clade have been circulating within Maricopa County for at least four years, making a case for endemicity. While the entrance of WNV into the United States occurred at the eastern coast of the United States in 1999, results from this study indicate that WNV strains circulating in the southwestern United States are now the most ancestral extant representatives from that introduction.

## Supporting information

S1 Table Sequence metadata in phylogenetic order. The Genbank accession number, the host or vector mosquito that the strain was isolated from, the state of isolation, isolation date to the nearest hundredths of a year, major WNV clade the strain belongs to, clade-defining amino acid substitutions, and notable convergent substitutions are included. We adopted the host/vector naming scheme as in Pybus et al.[14]:**Hosts:** Ah, *Ardea herodias* (blue heron); Bj, *Buteo jamaicensis* (red-tailed hawk); Bu, *Bonasa umbellus* (ruffed grouse); Bvs, *Butorides virescens* (green heron); Cb, *Corvus brachyrhynchos* (common crow); Cc, *Cyanocitta cristata* (blue jay); Ccs, *Cardinalis cardinalis* (northern cardinal); Cl, *Columba livia* (pigeon); Dc, *Dumetella carolinensis* (catbird); Ec, *Equus caballus* (horse); Fa, *Fulica Americana* (American coot); Hs, *Homo sapiens* (humans); Pc, *Phoenicopterus chilensis* (Chilean flamingo); Ph, *Pica hudsonia* (black-billed magpie); Pn, *Pica nuttalli* (yellow-billed magpie); Px, *Phalacrocorax sp*. (cormorant); Qq, *Quiscalus quiscula* (common grackle); Zm, *Zenaida macroura* (mourning dove); **Vectors:** Cn, *Culex nigripalpus* (mosquito); Cp, *Culex pipiens* (mosquito); Cq, *Culex quinquefasciatus* (mosquito); Cs, *Culex stigmatosoma* (mosquito); Ct, *Culex tarsalis* (mosquito); Cx, *Culex sp*. (mosquito). Dates were calculated using the collection dates’ corresponding day of the epoch calendar which was then divided by the total number of days in the year (365). The resulting decimal calculation was then added onto the collection year to the hundredths place.
(XLSX)

S2 Table Tiled amplicon primer pairs. Primer pairs, the amplification pools to which they belong, primer lengths, melting temperature (Tm), GC content, and start and end position in relationship to the NY99 strain are included. This information was directly output from Primal Scheme [7].
(XLSX)

S3 Table Model comparison results in duplicate from the generalized stepping stone sampling analyses. (XLSX)

S1 File Phylogenetically ordered amino acid alignment of the E protein. A valine at position 159 indicates the NY99 lineage, whereas an alanine indicates NA/WN02 or SW/WN03 lineages.
(FAS)

S2 File Phylogenetically ordered amino acid alignment of the NS4 protein. A threonine at position 85 indicates the SW/WN03 lineage.
(FAS)

S3 File Phylogenetically ordered amino acid alignment of the NS5 protein. A change from lysine to arginine at position 314 has occurred in several strains isolated in the southwestern United States, including all Arizona strains.
(FAS)

S4 File Phylogenetically ordered nucleotide alignment of the WNV coding region. (FAS)
